# *Mycobacterium bovis* BCG–Associated Osteomyelitis/Osteitis, Taiwan

**DOI:** 10.3201/eid2103.140789

**Published:** 2015-03

**Authors:** Nan-Chang Chiu, Meng-Chin Lin, Wen-Li Lin, Shin-Yi Wang, Hsin Chi, Li-Min Huang, Ren-Bin Tang, Yhu-Chering Huang, Ching-Chuan Liu, Fu-Yuan Huang, Tzou-Yien Lin

**Affiliations:** Mackay Memorial Hospital, Taipei, Taiwan (N.-C. Chiu, M.-C. Lin, W.-L. Lin, H. Chi, F.-Y. Huang);; Mackay Junior College of Medicine, Nursing and Management, Taipei (N.-C. Chiu, H. Chi);; Taiwan Centers for Disease Control, Taipei (S.-Y. Wang);; National Taiwan University Hospital, Taipei (L.-M. Huang);; Cheng Hsin General Hospital, Taipei (R.-B. Tang);; Chang Gung Memorial Hospital, Taoyuan, Taiwan (Y.-C. Huang, T.-Y. Lin);; National Cheng Kung University Hospital, Tainan, Taiwan (C.-C. Liu);; Ministry of Health and Welfare, Executive Yuan, Taipei (T.-Y. Lin)

**Keywords:** Mycobacterium bovis, BCG, osteomyelitis, osteitis, Taiwan, tuberculosis and other mycobacteria, vaccine injury compensation program, bacteria

**To the Editor:** Thirty-eight patients with *Mycobacterium bovis* BCG–associated osteomyelitis/osteitis, including 8 who were previously reported (*1*), were identified during Taiwan’s vaccine injury compensation program during 1989–2012; a total of 30 (79%) patients applied for compensation during 2009–2012 (Figure). In Taiwan, a laboratory program to differentiate BCG from other species of the *M. tuberculosis* complex, using a kit for the Tokyo-172 vaccine strain spoligotyping, was established in 2004 (*1*). Since 2008, the isolated extrapulmonary tuberculosis strains and pathologic specimens collected from children <5 years of age have been sent to the national reference mycobacterial laboratory for BCG detection (*2*). The detected incidence of BCG osteitis/osteomyelitis increased from 3.68 cases per million vaccinations during 2002–2006 to 30.1 per million during 2008–2012.

**Figure:**
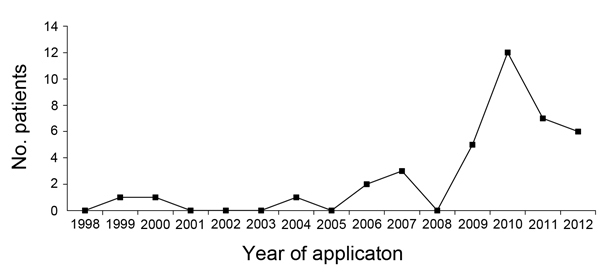
Number of patient applications for compensation as a result of *Mycobacterium bovis* BCG osteomyelitis/osteitis to vaccine injury compensation program, Taiwan, 1998–2012.

Parents or guardians signed written consent forms on behalf of the children when they submitted claims for the vaccine injury compensation program. After consent, children’s hospital information was stored in the Taiwan Centers for Disease Control database and used for research.

Of the 38 compensated BCG osteomyelitis/osteitis patients, 18 were boys. According to chart review, no patients had immunodeficiency or other underlying conditions; however, 3 were premature babies (born at 34–36 weeks of gestation). Eighteen (47%) children had received BCG at <1 week of age, 12 (32%) at 1–4 weeks, 7 (18%) at 1–2 months, and 1 at >2 months. The average age at inoculation was 16.2 ± 16.6 days. Symptoms or signs began 3–32 months (average 12.4 ± 6.1 months) after BCG vaccination; for 68%, symptoms or signs developed 7–18 months after vaccination (Technical Appendix Figure). Time from vaccination to onset of symptoms or signs did not differ for the 3 premature infants.

As in previous reports (*3*,*4*), extremity bones were more commonly involved than axial bones. For 30 (79%) children, extremity bones were involved: 14 right lower limbs, 7 left lower limbs, 6 left upper limbs, and 3 right upper limbs. The tibia was the most common site (9 patients), followed by ankle bones (8 patients), femur (4 patients), radius and thumb (3 patients each), humerus and knee (2 patients each), and ulna (1 patient). Of these, 2 patients had 2 bony lesions. In 8 (21%) children, axial bones were involved: 5 sternums, 2 thoracic vertebrae, and 1 right rib. Presentation included a mass (25 [66%] children), tenderness (22 [58%]), limping (19 [50%]), redness (14 [37%]), and heat (7 [18%]). Average time from first clinical visit to final surgical management was 1.6 ± 2.1 months.

Eight (53%) of 15 patients had positive tuberculin skin test results. No specific abnormalities were found with regard to blood cell counts and inflammation markers or to chest radiographs, except for 1 child with rib erosion. Pathologic diagnosis of *Mycobacterium* infection from bony specimens was recorded for 35 (92%) patients. For 29 (76%), diagnosis was conducted by molecular study, including 25 (66%) by the national reference mycobacterial laboratory. For 4 patients, diagnosis was confirmed by culture of *M. bovis*. Osteomyelitis/osteitis for 5 patients was considered BCG related according to pathologic diagnosis of *Mycobacterium* infection, BCG vaccination history, and lack of a history of contact with a person with tuberculosis.

Thirty-two (84%) children underwent surgery (excision, debridement, open biopsy), 4 children received arthrotomy (3 ankle and knee joint), and 2 children underwent only aspiration biopsy. All patients received isoniazid and rifampin therapy; 33 patients also received pyrazinamide, and 6 received additional ethambutol therapy. Medications were adjusted after diagnoses changed from tuberculosis to BCG infection. Two patients had major sequelae, both involving the thoracic spine and causing severe kyphosis.

Adverse reactions after BCG vaccination depend on the BCG dose, vaccine strain, vaccine administration method, injection technique, and recipient’s underlying immune status (*5*). The vaccine strain and manufacturing process in Taiwan did not change during the study period. Findings were not associated with a specific batch of vaccine, inoculation age, underlying disease, or *Salmonella* spp. infection. Patients had no common birth place, hospital, or area of residence. We believe the increased number of cases resulted mainly from policy changes and laboratory facility improvements.

A surgical approach to obtain a specimen is indicated. However, because medical treatment usually yields a good outcome (*6*), extensive debridement should be avoided. Although some patients with lower extremity involvement initially limped, most were able to walk well later. Vertebral involvement is rare. Unlike previously reported cases (*7*,*8*), both patients reported here who had vertebral involvement had sequelae. For young children with suspected vertebral tuberculosis but no tuberculosis contact history, a biopsy specimen for BCG studies is preferable to spondylectomy. Although no definite immunologic deficit was found in these BCG osteomyelitis/osteitis patients, 2 other compensated infants with disseminated BCG during the same period in Taiwan had identified immunodeficiency (*9*). Studies are ongoing by the Taiwan Centers for Disease Control to evaluate medical treatment duration, long-term outcomes, and more detailed immune genetic tests.

Technical Appendix Interval between *Mycobacterium bovis* BCG inoculation and osteomyelitis/osteitis onset in 38 vaccine injury compensation program patients, Taiwan, 1998–2012.
