# Supplementary material for: Mycobacterium bovis BCG–Associated Osteomyelitis/Osteitis, Taiwan
Source: Emerg Infect Dis. 2015 Mar;21(3):539–40. doi: 10.3201/eid2103.140789 (PMC4344265; doi:10.3201/eid2103.140789)
Supplement: Technical Appendix — Interval between Mycobacterium bovis BCG inoculation and osteomyelitis/osteitis onset in 38 vaccine injury compensation program patients, Taiwan, 1998–2012. [file 14-0789-Techapp-s1.pdf]

Article DOI: http://dx.doi.org/10.3201/eid2103.140789

Mycobacterium bovis BCG–Associated Osteomyelitis/Osteitis, Taiwan

Technical Appendix

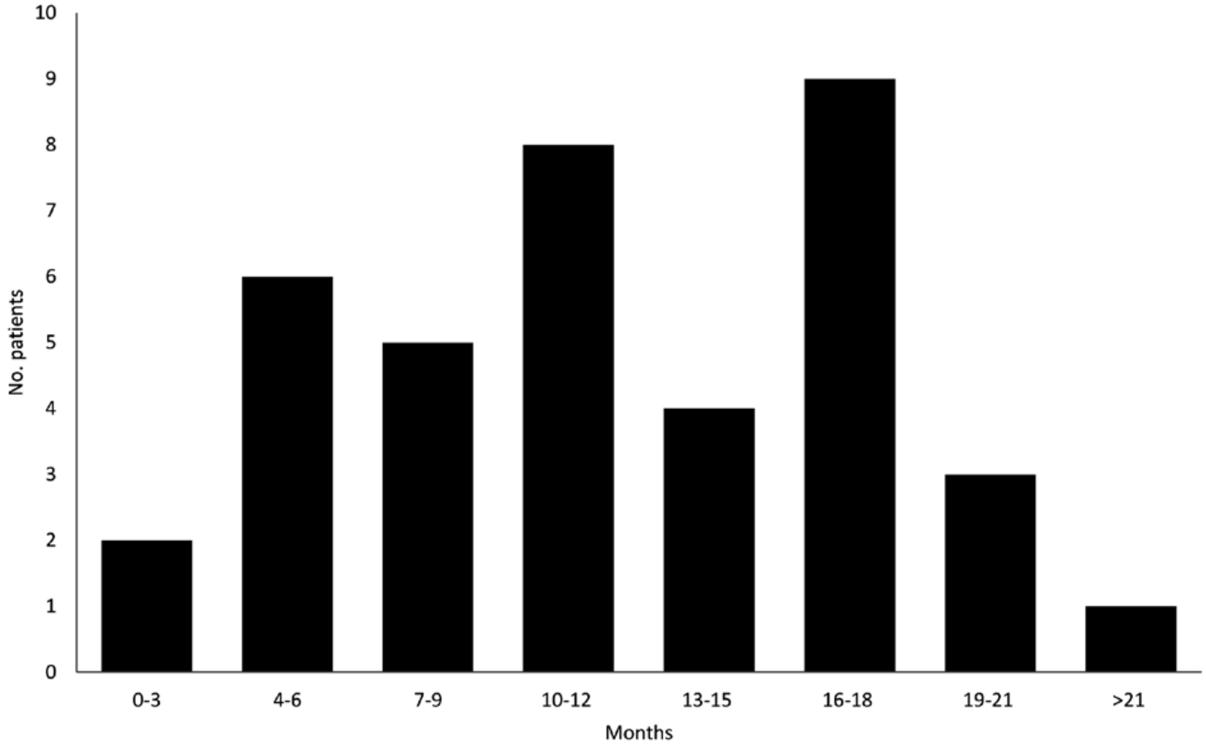

Technical Appendix Figure. Interval between Mycobacterium bovis BCG inoculation and osteomyelitis/osteitis onset in 38 vaccine injury compensation program patients, Taiwan, 1998–2012.
